# Efficacy of biologic DMARDs in improving the clinical response of patients with polyarticular juvenile idiopathic arthritis: a meta-analysis of RCTs

**DOI:** 10.1186/s42358-025-00488-x

**Published:** 2025-11-10

**Authors:** Pedro Henrique Aquino Gil de Freitas, Mylena Maria Guedes de Almeida, Áurea Maria Salomão Simão, Ana Beatriz Bertol, Barkhá Vijendra, Bianca Lisa de Faria, Camila Maria Paiva França Telles

**Affiliations:** 1 Faculty of Medicine, Federal University of Amazonas, Manaus, Brazil; 2 Faculty of Medicine, Federal University of Minas Gerais, Belo Horizonte, Brazil; 3 Faculty of Medical Sciences of Santa Casa de São Paulo, São Paulo, Brazil; 4 University of South Santa Catarina, Florianópolis, Brazil; 5 Eduardo Mondlane University, Maputo, Mozambique; 6 Instituto da Criança do Hospital das Clínicas da Universidade de São Paulo, São Paulo, Brazil; 7 Rua José de Arimateia, 759, apto. 43, Adrianopolis, Manaus, AM 69060-081 Brazil

**Keywords:** Polyarticular juvenile idiopathic arthritis, Biologic DMARDs, Clinical response

## Abstract

**Background:**

We aimed to conduct a meta-analysis of randomized controlled trials (RCTs) to examine the efficacy of biologic DMARDs in improving the clinical response of patients with polyarticular JIA.

**Methods:**

The literature and relevant reviews were searched for published clinical studies comparing the efficacy of standard therapy without biologic DMARDs to that of biologic DMARDs in patients with polyarticular JIA. The focus was on the minimal clinical effectiveness criteria of the pediatric American College of Rheumatology (pedACR30–100), time to disease flare, remission clinical, inactive disease, and pedACR30/flare. This meta-analysis followed the Preferred Reporting Items for Systematic Reviews and Meta-Analysis (PRISMA) guidelines.

**Results:**

In total, 9 randomized controlled trials were included in the study. Compared with standard therapy, treatment with biologics significantly improved PedACR70 (relative risk [RR] 1.68; 95% confidence interval [CI] 1.43–1.96; *p* < 0.00001). Similarly, PedACR30 (RR 1.37; CI 1.19–1.58; *p* < 0.0001), PedACR50 (RR 1.49; CI 1.25–1.77; *p* < 0.00001), PedACR90 (RR 1.67; CI 1.34–2.09; *p* < 0.00001) and PedACR100 (RR 1.88; CI 1.05–3.35; *p* = 0.03) were also significantly improved with biologic treatment. However, patients on standard therapy had worse flare outcomes, as determined by the PedACR30/Flare (RR 0.56; CI 95% 0.45–0.70; *p* < 0.00001). Additionally, the time to disease flare was significantly shorter with standard therapy (hazard ratio [HR] 0.38; 95% CI 0.27–0.54; *p* < 0.00001).

**Conclusion:**

Compared with standard therapy, biologic DMARDs lead to significant and long-term improvements in signs and symptoms in polyarticular JIA patients.

**Trial registration:**

This meta-analysis was registered in PROSPERO under the registration number CRD42023494938 on December 18, 2023.

**Supplementary Information:**

The online version contains supplementary material available at 10.1186/s42358-025-00488-x.

## Background

Juvenile idiopathic arthritis (JIA) is a heterogeneous group of rheumatic diseases with onset by the age of 16 years, as defined by the International League Against Rheumatism (ILAR) [1, 2]. JIA subtypes have distinct pathogeneses that are not completely understood and involve immunological changes, inflammatory cytokines, and autoantibody production, causing articular inflammation of the synovium [3]. JIA affects an estimated 2 million children worldwide, making it one of the most prevalent pediatric diseases [4]. For various categories of JIA, distinct recommendations exist regarding patient management and treatment regimens. Polyarticular juvenile idiopathic arthritis (PolyJIA) represents a heterogeneous group of children with polyarthritis (≥ 5 joints at any time), excluding those with systemic arthritis, sacroiliitis, or extra-articular manifestations such as psoriasis, uveitis, or inflammatory bowel disease [5].

The treatment of PolyJIA includes nonsteroidal inflammatory drugs, intraarticular injections of glucocorticoids, systemic glucocorticoids, conventional synthetic disease-modifying antirheumatic drugs (DMARDs), or biological DMARDs [5–7]. Conventional or nonbiological DMARDs include methotrexate (MTX), leflunomide, and sulfasalazine. The current recommendation favors methotrexate over leflunomide and sulfasalazine [5]. However, some patients are intolerant of MTX side effects, which justify the use of leflunomide or sulfasalazine [8–10]. Biologic DMARDs are used in patients refractory to MTX treatment or other standard therapies. ^5^. Research on the treatment of PolyJIA with biologic DMARDs, which started in the 2000s, revealed improvements in long-term outcomes [11–19]. 

Additionally, more than half of children with any type of JIA may evolve to receive biologic DMARDs [20, 21]. Some randomized clinical studies have compared the efficacy and effectiveness of biologic DMARDs with those of standard treatments (methotrexate +/- glucocorticoids) in the management of PolyJIA; however, there is still a gap in the literature regarding this therapy [11–19]. 

A previous meta-analysis addressed this topic, but it suffered from a sparseness of studies and included studies with different populations and outcomes [23]. In addition, recent studies have investigated the efficacy and other outcomes of clinical interest, such as the time to disease flare. Thus, this systematic review and meta-analysis were conducted to quantitatively evaluate the use of biologic DMARDs in PolyJIA.

## Methods

The analysis adhered to the Preferred Reporting Items for Systematic Reviews and Meta-analysis (PRISMA) guidelines and was registered in PROSPERO under the registration number CRD42023494938 [22]. 

### Eligibility criteria

The inclusion criteria for the meta-analysis on PolyJIA were as follows: (1) randomized clinical trials comparing biological DMARDs and standard therapy, with a diagnosis according to either the International League of Associations for Rheumatology (ILAR) conference in 2001 or the American College of Rheumatology (ACR) conference in 1986; (2) studies published in the English language; and (3) studies that reported outcomes of interest; (4): pedACR30, pedACR50, pedACR70, pedACR90, pedACR100, time to disease flare, remission clinical, inactive disease and pedACR30 flare. The exclusion criteria included (1) duplicate studies, (2) overlapping populations, and (3) incomplete studies or conference abstracts.

### Literature search

The search was performed in December 2023 on PubMed, Embase, and Cochrane Central for RCTs comparing the efficacy of standard therapy without biologic DMARDs to that of biologic DMARDs in patients with PolyJIA. The PubMed search strategy and research terms were predefined (Supplementary Table 1S). In cases where results or publications were not available, the study authors were contacted.

### Study selection, data collection process, and risk of bias assessment

Two authors (PF and MA) developed and tested a data extraction sheet, searched databases using predefined search terms, screened titles and abstracts, conducted a full-text review of selected studies, and extracted relevant data. Any disagreements regarding study inclusion were resolved through consensus, with a third author (AS) consulted if consensus was not reached. The literature quality was assessed through the Cochrane Collaboration tool RoB-2 tool [23]. The assessment considered factors such as the random allocation method, allocation concealment, use of blinding for participants, completeness of result data, presence of selective reporting, and other potential biases.

### Statistical analysis

The meta-analysis was conducted via RevMan 5.4 software, which is recommended by the Cochrane Collaboration. Efficacy and safety statistics were measured via risk ratios (RRs) or hazard ratios (HRs) along with their respective 95% confidence intervals (CIs). The Cochrane Q test and I2 statistic were employed to investigate heterogeneity, with I2 > 40% considered significant for heterogeneity. The data were combined and analyzed via a random effects model for all outcomes. The intention-to-treat analysis was selected to minimize bias and ensure standardization in the study.

### Outcomes

The primary outcome of interest was (1) PedACR70, which measures 70% improvement in the American College of Rheumatology (ACR) criteria for assessing treatment response in pediatric patients. The secondary endpoints included (2) PedACR30, (3) PedACR50, (4) PedACR90, (5) PedACR100, (6) clinical remission, which refers to the absence of disease activity; (7) inactive disease, which indicates the absence of active disease symptoms; (8) PedACR30/Flare, which measures the incidence of disease flares in patients who achieved PedACR30; (9) time to disease flare, which measures the time until a disease flare occurs; and (10) adverse events, which are classified according to Hartwig’s Severity Assessment Scale (HSAS) [24].

## Results

### Study selection

**Fig. 1:**
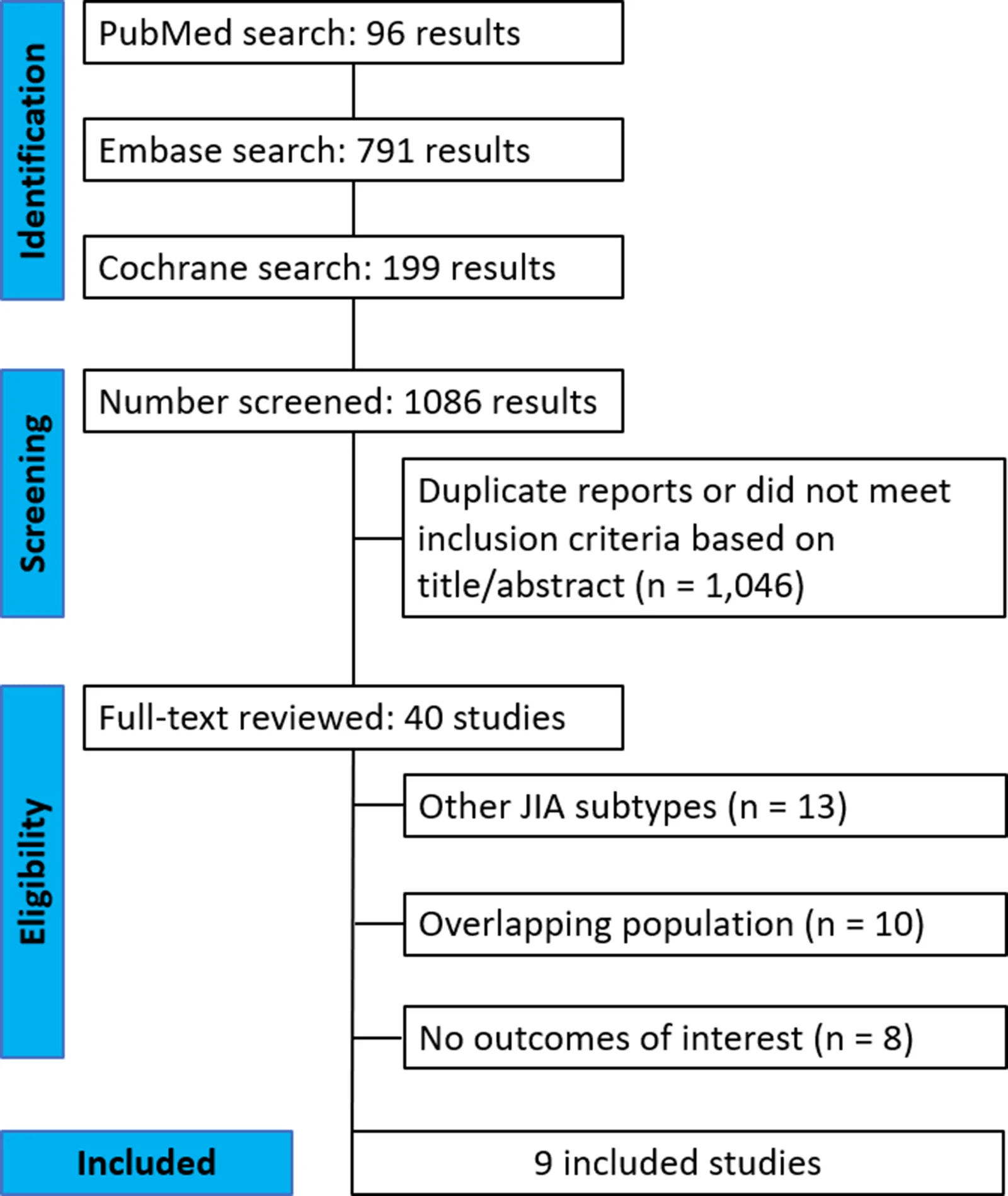
PRISMA flow diagram of study screening and selection

The search retrieved 1086 studies. Among these, 1046 (96.3%) were duplicates, did not meet the inclusion criteria on the basis of their title or abstract, or were excluded from the full-text screening because of the approach to other JIA subtypes, overlapping populations, or no outcomes of interest. Nine studies met the inclusion criteria for this review and were included in the analysis (Fig. 1).

### Study characteristics

Table 1 details the nine randomized clinical studies included in this meta-analysis. In total, this study included 803 participants, all of whom were diagnosed with JIA. One study evaluated two distinct intervention groups [12].

**Table 1:** Baseline characteristics of included studies

Study	Intervention	Control	No. Of patients: Intervention/Control	Age (y): Intervention/Control	Percentage of female patients: Intervention/Control
Alexeeva, 2021 [15]	Etanercept + MTX	Placebo + MTX	35/33	9.8/6.62§	64.71%/74.76%
Brunner, 2014 [14]	Tocilizumab	Placebo	82/81	NR	NR
Brunner, 2017 [19]	Golimumab	Placebo	78/76	11.1/11.1†	75.6%/75%
Lovell, 2000 [11]	Etanercept	Placebo	25/26	NR	76%/58%
Lovell, 2008* [12]	Adalimumab + MTX	Placebo	38/37	11.7/10.8†	79%/81%
Lovell, 2008 [12]	Adalimumab	Placebo	30/28	11.1/11.3†	77%/71%
Ruperto, 2007 [17]	Infliximab + MTX	MTX + Placebo	58/59	11.3/11.1†	83.3%/79%
Ruperto, 2008 [18]	Abatacept	Placebo	60/62	12.3/12†	72%/73%
Ruperto, 2021 [16]	Tofacitinib	Placebo	72/70	13/13§	75%¶
Wallace, 2012 [13]	Etanercept + MTX + Prednisolone	MTX + Placebo	42/43	9.9/11.1†	69%/79.1%

*Intervention Arm Adalimumab + Methotrexate; MTX – Methotrexate; § IQR range; †Mean SD; ¶Total population; NR – Not Reported

### Quality assessment results

The risk of bias within individual studies was assessed via the ROB-2 tool. All of the included studies reported a low risk of bias, and one had some concerns due to deviations from the intended intervention [11] (Supplementary Fig. S1).

### PedACR 30

We identified eight studies that assessed PedACR30. The meta-analysis revealed that biologic treatment significantly increased the likelihood of reaching PedACR30 compared with standard therapy (RR 1.37; CI 1.19–1.58; *p* < 0.0001; I²=42%) (Supplementary Fig. S2).

### PedACR 50

Eight studies assessed PedACR50. Compared with standard therapy, biologic treatment significantly increased the likelihood of meeting the PedACR50 (RR 1.49; CI 1.25–1.77; *p* < 0.00001; I²=47%) (Supplementary Fig. S3).

.

### PedACR 70

**Fig. 2:**
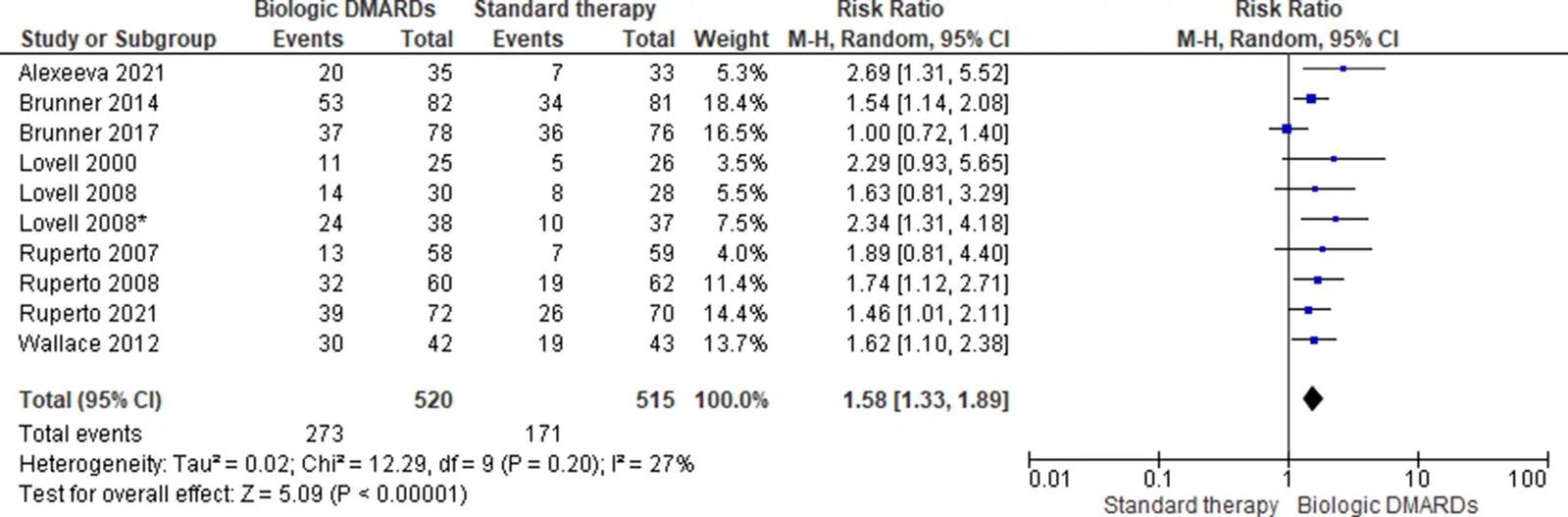
PedACR70. *Intervention Arm Adalimumab + Methotrexate

The analysis of nine studies that investigated treatment with biologics revealed significantly greater PedACR70 values than did standard therapy (RR 1.68; 95% CI 1.43–1.96; *p* < 0.00001; I²=0%) (Fig. 2).

### PedACR 90

**Fig. 3:**
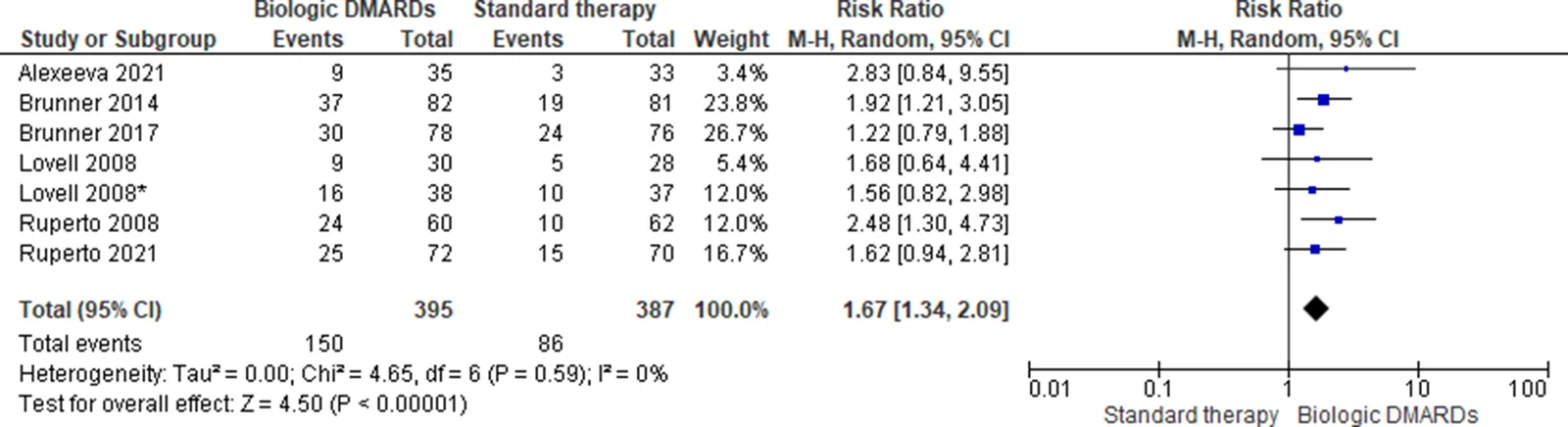
PedACR90. *Intervention Arm Adalimumab + Methotrexate

Six studies examined PedACR90. Compared with standard therapy, treatment with biologics significantly improved PedACR90 (RR 1.67; CI 1.34–2.09; *p* < 0.00001; I²=0%) (Fig. 3).

### PedACR 100

**Fig. 4:**
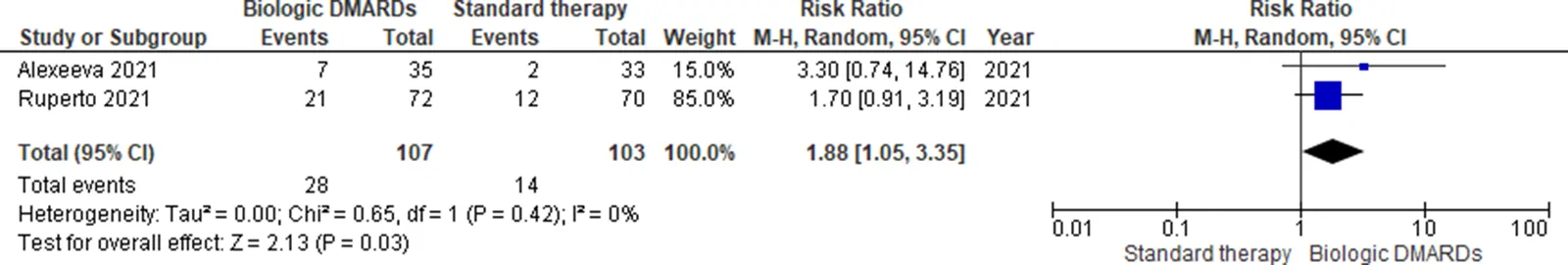
PedACR100

Two studies reported PedACR100. The pooled meta-analysis revealed that biologics significantly improved the achievement of PedACR100 compared with standard therapy (RR 1.88; CI 1.05–3.35; *p* = 0.03; I²=0%) (Fig. 4).

### Clinical remission

Four studies assessed clinical remission, which was defined as the absence of disease activity. The meta-analysis revealed no significant difference in achieving clinical remission between biologics and standard therapy (RR 1.36; 95% CI: 0.83–2.22; *p* = 0.22; I² = 0%) (Supplementary Fig. S4).

### Inactive disease

Five studies reported the outcome of inactive disease, defined as the absence of active disease symptoms. The meta-analysis revealed no significant difference in achieving inactive disease when biologics were compared with standard therapy (RR 1.57; CI 0.99–2.51; *p* = 0.06; I²=67%) (Supplementary Fig. S5).

### PedACR30/Flare

**Fig. 5:**
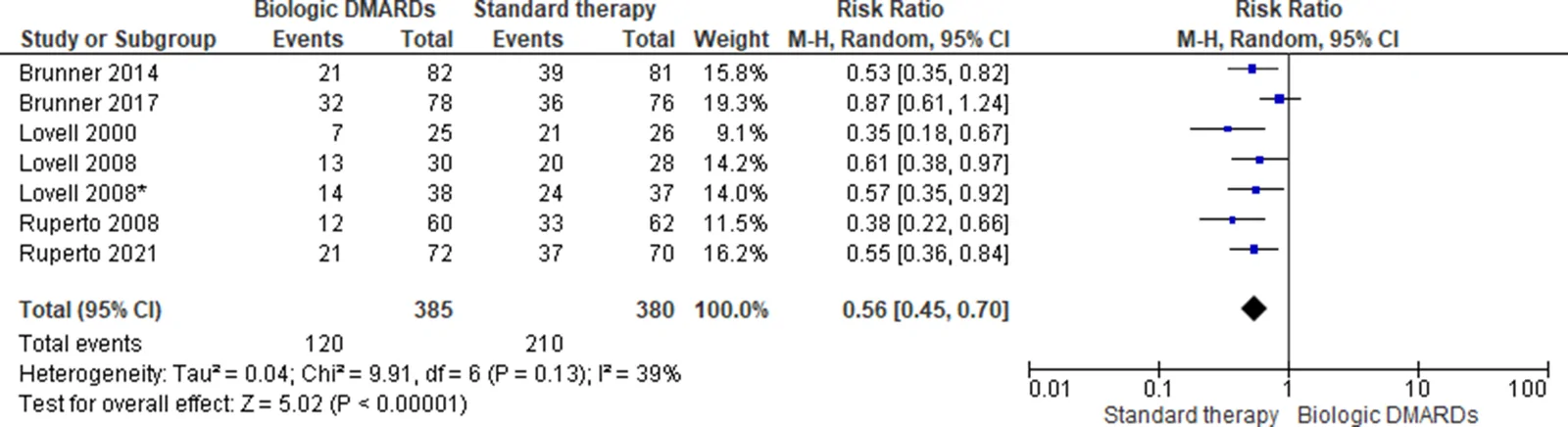
PedACR30/Flare. *Intervention Arm Adalimumab + Methotrexate

We identified six studies that assessed PedACR30/Flare. The flare rate was significantly lower with biologics than with standard therapy (RR 0.56; CI 95% 0.45–0.70; *p* < 0.00001; I²=39%) (Fig. 5).

### Time to disease flare

**Fig. 6:**
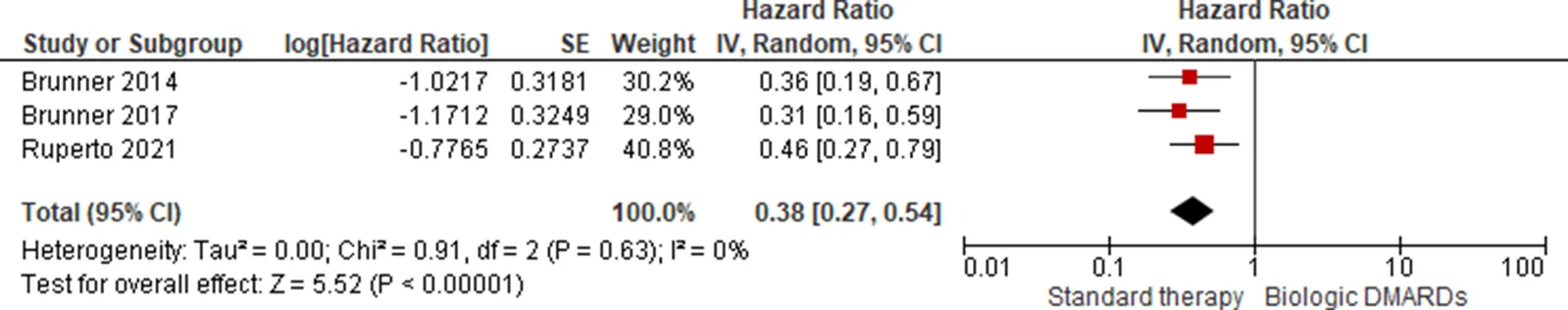
Time to disease flare

The time to disease flare was significantly shorter in the standard therapy group than in the biologics group (HR 0.38; 95% CI 0.27–0.54; *p* < 0.00001; I²=0%) (Fig. 6).

### Adverse events

All nine studies analyzed adverse events (AEs) via various methods. Consequently, a systematic review will be performed without numerical analysis. No fatalities were recorded in any of the studies. A summary table was constructed to synthesize the main results of the included studies (Table 2).

Lovell et al. [11] reported one AE classified as moderate according to Hartwig’s Severity Assessment Scale (HSAS) and two as severe in the intervention group with the use of etanercept, whereas the remaining AEs were classified as mild in both groups.

Lovell et al. [12] reported 308 AEs in the placebo group, of which only one was classified as severe according to the HSAS, specifically, gastroduodenitis. The remaining AEs included seventy-seven related to injection-site reactions, eleven cases of nasopharyngitis, eleven upper respiratory tract infections, and seven viral infections, all of which were classified as mild. In the adalimumab plus methotrexate group, all AEs were classified as mild, including seventy-three related to injection-site reactions, five related to nasopharyngitis, six related to upper respiratory tract infections, and seven related to viral infections. Similarly, in the adalimumab therapy group, all AEs were classified as mild, with seventy-one related to injection-site reactions, six upper respiratory tract infections, and eight viral infections.

Wallace et al. [11]reported five AEs classified as severe according to the HSAS in the etanercept group, with none reported in the placebo group. Additionally, eighteen infections occurred during 247 months of methotrexate (MTX) monotherapy (0.87 per year), whereas 17 infections occurred during 360 months of combined etanercept, MTX, and prednisolone therapy (0.57 per year).

Brunner et al. [14] reported three severe adverse events in both groups, with the remaining events classified as moderate or mild. In the tocilizumab group, fourteen cases of nasopharyngitis, four cases of upper respiratory infections, and three cases of pharyngitis were observed. In the placebo group, there were nine cases of nasopharyngitis, two cases of upper respiratory infections, and three cases of pharyngitis.

Brunner et al. [19] reported that sixty-one patients in the golimumab group experienced at least one adverse event, including seven cases of serious reactions, three of which involved worsening of JIA. The most common AEs were thirty-seven cases of upper respiratory tract infections and thirteen cases of nasopharyngitis. In the placebo group, sixty-three patients developed at least one AE, including nine patients with serious reactions, five of whom experienced worsening of JIA. The most common AEs were forty-eight cases of upper respiratory tract infections and twenty-one cases of nasopharyngitis.

Alexeeva et al. reported only five AEs in the etanercept plus methotrexate group, including two infections, whereas the placebo group had three adverse events, all related to infections. All AEs in both groups were classified as moderate or mild.

Ruperto et al. [17] reported that fifty-eight patients in the infliximab group experienced adverse events, with nineteen classified as serious. The primary cause was infection, accounting for forty-one cases. Two patients discontinued the study because of infusion reactions, but no deaths were reported. In the placebo group, forty-nine patients experienced AEs, with only three classified as serious. Infections were also the main cause, with twenty-eight cases. One patient discontinued the study because of circulatory failure.

Ruperto et al. [18] reported thirty-seven AEs in the abatacept group, none of which were serious. The remaining events were classified as moderate or mild, including four cases of nasopharyngitis and four upper respiratory infections. In the placebo group, thirty-four AEs were reported, including two severe events (one hematoma and one case of varicella with encephalitis), three cases of nasopharyngitis and five upper respiratory infections.

Ruperto et al. [16] reported sixty-eight AEs in the tofacitinib group, one severe, nine moderate and fifty-eight mild, with thirteen cases of upper respiratory tract infections. In the placebo group, sixty-three AEs were recorded, five severe, eight moderate and fifty mild, including nine cases of upper respiratory tract infections.

Importantly, no cases of tuberculosis were reported in any of the included studies, despite being a well-known and serious adverse event associated with immunosuppressive therapies such as biologic DMARDs.

**Table 2:** Adverse events of included studies

Study	Intervention	Total AEs – DMARD Group	Severe AEs – DMARD Group	Total AEs – Placebo Group	Severe AEs – Placebo Group
Alexeeva, 2021 [15]	Etanercept + MTX	5	0	3	0
Brunner, 2014 [14]	Tocilizumab	147	3	121	3
Brunner, 2017 [19]	Golimumab	NR	7	NR	9
Lovell, 2000 [11]	Etanercept	NR	0	NR	0
Lovell, 2008* [12]	Adalimumab + MTX	234	0	155	1
Lovell, 2008 [12]	Adalimumab	171	0	153	0
Ruperto, 2007 [17]	Infliximab + MTX	58	19	49	3
Ruperto, 2008 [18]	Abatacept	37	0	34	2
Ruperto, 2021 [16]	Tofacitinib	68	1	63	5
Wallace, 2012 [13]	Etanercept + MTX + Prednisolone	NR	5	NR	0

*Intervention Arm Adalimumab + Methotrexate; MTX – Methotrexate; AEs – Adverse events; NR – Not Reported

## Discussion

This systematic review and meta-analysis explored the efficacy of biological DMARDs in children with PolyJIA. The findings of our meta-analysis suggest that biological drugs are safe and efficient treatment options for polyarticular juvenile idiopathic arthritis. More than half of the patients included in our meta-analysis achieved 70% improvement with biological drugs. Our meta-analysis revealed that patients treated with biologic DMARDs experienced fewer disease flares and increased in duration until a flare occurred. We did not find any significant differences between etanercept, adalimumab, tocilizumab, tofacitinib, or baricitinib in terms of efficacy outcomes. Our findings are consistent with those of a previous meta-analysis, which concluded that abatacept, adalimumab, etanercept, and tocilizumab were equally effective in preventing disease flares in polyarticular JIA, with a low frequency of serious adverse events (SAEs) across all biologic agents [25]. In addition, our study incorporates more recent trials, including those evaluating tofacitinib and golimumab. These findings are in line with previous evidence [26, 27].

One of the major concerns regarding the use of biologic DMARDs is the potential adverse effects resulting from the immunosuppressive nature of these treatments. In our meta-analysis, injection site reactions were the most common AE. Infections were observed in both groups with similar frequencies, although the results were reported in various ways. Eighty-seven patients in the intervention group and eighty-four in the placebo group presented infections. Serious adverse events occurred in eight patients in the intervention group and seven in the placebo group. No withdrawals due to adverse events in the double-blind phases of the studies were reported, and no deaths related to biological DMARD therapy were reported. Etanercept appears to be safer than the other agents. These findings are consistent with published research on the safety of these medications in children [28, 29]. In addition, real side effects are evaluated in phase IV studies.

Biological DMARDs are first-line treatment alternatives for polyJIA patients who are intolerant to or have an ineffective response to methotrexate [5]. These drugs are associated with better disease control, fewer disease flares, and increased time between flares. Additionally, they significantly improve patients’ quality of life, pain, sleep quality, and functional ability [30–34]. Nevertheless, the cost of such medications can be a barrier to access. The incremental cost-effectiveness ratios versus methotrexate for adalimumab, etanercept, tocilizumab, and abatacept are £38,127, £32,256, £38,656, and £39,536 per quality-adjusted life year, respectively [35]. This value represents the increase in the overall cost of living for these patients. Such high costs represent a significant barrier to access for many patients, particularly in low- and middle-income countries, and may limit the real-world applicability of these therapies despite their demonstrated efficacy.

This meta-analysis has certain limitations. Importantly, different methods for including patients with different criteria for polyarticular juvenile idiopathic arthritis exist, as some studies suggest the addition of other forms to this classification. This heterogeneity in inclusion criteria may have introduced clinical variability among study populations, thereby affecting the homogeneity of the pooled results. Some studies could not be included in this meta-analysis because of heterogeneous subtypes in patient analysis. Currently, the ILAR classification is the most widespread and used in this study, but others, such as the PRISTO, have different criteria [1, 2]. In addition, new perspectives from genetic studies suggest that systemic JIA and other subtypes may be different diseases with specific pathophysiologic pathways [36]. Another limitation is the lack of standardization in analyzing the adverse effects of these drugs. Finally, the external validity of this study is low, as the sample size is relatively small to obtain an analysis that represents the general population of patients, for example, the impact of DMARD use in patients with long disease durations. Although 803 patients were included, this number may still be insufficient to account for the wide clinical heterogeneity and treatment responses observed in the broader population of PolyJIA patients.

## Conclusion

This systematic review and meta-analysis revealed that compared with standard therapy, biological DMARD therapy is effective at achieving 70% improvement in PedACR criteria, reducing disease flares, and increasing the time between flares, with unremarkable differences in the occurrence of adverse effects. These findings may support rheumatologists in their clinical judgment during practice. Further research, considering standardized classification criteria, is necessary to gather more unbiased evidence.

## Supplementary Information

Below is the link to the electronic supplementary material.

Supplementary Material 1

## Data Availability

The data supporting the findings of this study are available within the manuscript and its supplementary information files.

## Data availability

The data underlying the findings of this study will be made available upon reasonable request to the corresponding author.
