# Supplementary material for: Efficacy of biologic DMARDs in improving the clinical response of patients with polyarticular juvenile idiopathic arthritis: a meta-analysis of RCTs
Source: Adv Rheumatol. 2025 Nov 10;65:55. doi: 10.1186/s42358-025-00488-x (PMC13488648; doi:10.1186/s42358-025-00488-x)
Supplement: Supplementary file 1 [file 42358_2025_488_MOESM1_ESM.docx]

**Supplementary file**

**Supplementary Table S1:** Detailed search strategy used for all databases ......................................... 2

**Supplementary Fig. S1:** Quality assessment of randomized-controlled-trials through Rob 2 tool ...... 3

**Supplementary Table S2:** Abbreviation key ......................................................................................... 4

**Supplementary Fig. S2:** PedACR50 ....................................................................................................... 5

**Supplementary Fig. S3:** PedACR90 ....................................................................................................... 5

**Supplementary Fig. S4:** Clinical Remission ........................................................................................... 5

**Supplementary Fig. S5:** Inactive Disease .............................................................................................. 5

**Supplementary Table S1:** Detailed search strategy used for all databases

| **Search number** | **Terms** |
| --- | --- |
| **#1** | ("polyarticular juvenile idiopathic arthritis" OR "juvenile rheumatoid arthritis" OR "juvenile idiopathic arthritis") |
| **#2** | ("Biologic disease-modifying anti-rheumatic drugs" OR Adalimumab OR Infliximab OR Etanercept OR Abatacept OR Tocilizumab OR Tofacitinib OR Baricitinib OR Anakinra OR Canakinumab OR Golimumab OR Rituximab OR Rilonacept OR "biologic drugs" OR "biologic agents") |
| **#3** | (Placebo) |
| **#4** | #1 AND #2 AND #3 |

**Supplementary Figure S1:** Quality assessment of randomized-controlled-trials through Rob 2 tool

**Supplementary Table S2:** Abbreviation key:

| DMARDs | Disease-Modifying Antirheumatic Drugs |
| --- | --- |
| JIA | Juvenile İdiopathic Arthritis |
| ILAR | International League Against Rheumatism |
| PolyJIA | Polyarticular Juvenile İdiopathic Arthritis |
| MTX | Methotrexate |
| ACR | American College Of Rheumatology |
| PedACR30 | Pediatric American College of Rheumatology criteria 30% response |
| PedACR50 | Pediatric American College of Rheumatology criteria 50% response |
| PedACR70 | Pediatric American College of Rheumatology criteria 70% response |
| PedACR90 | Pediatric American College of Rheumatology criteria 90% response |
| PedACR100 | Pediatric American College of Rheumatology criteria 100% response |
| RR | Relative Risk |
| HSAS | Hartwig's Severity Assessment Scale |
| CI | Confidence interval |

**Supplementary Fig. S2: PedACR30**

**Supplementary Fig. S3:** PedACR50

**Supplementary Fig. S4:** Clinical Remission

**Supplementary Fig. S5:** Inactive Disease
